# Mr. Grose, on the Medicinal Use of Yeast

**Published:** 1800-02

**Authors:** J. H. Grose

**Affiliations:** Winslow


### *To the Editors of the Medical and Phyfical Journal.*

Gentlemen,

NOTHING, in my opinion, can be more ferviceable to the community at large, than laying before them a method of obviating complaints which have caufed the greateft defolation, and which at this feafon of the year are more immediately prevalent. It is a duty which every humane being muft feel fatisfied in performing; and though our fervices may not meet with that attention they deferve, the confcioufnefs of having difplayed the means, will, of itfelf, be a fufficient recompence.

In May, 1798, the child of John J———, (then refident at Coventry, but now an inhabitant of S———y,) aged two years, was attacked with a fevere pain in the head, and cough, which, by diminifhing his appetite, and his parents being unable to procure him proper support, reduced him to a very weak condition. Medical advice was obtained, and but little benefit derived; the cough still continued, and a gradual wasting of the body ensued. Providentially for the helpless sufferer, the father was informed by a clergyman, who occasionally practised physic, of the salutary effects of yeast, (or, as it is stiled here, barm,) in such cases; this medicine having been administered by him to several infants in a similar situation, and with the most beneficial effects.

They consented to try it, and the child was to take three tea spoonfulls night and morning. For the first week, no apparent advantage was derived; the body was rather purged, and they feared it would weaken him too much. The diet was simple, milk or broths, for his appetite was small; though he was occasionally permitted to have a little raisin wine diluted with water.

Towards the end of the second week, the cough considerably abated, the colour became more diffused, the purging was inconsiderable, and his appetite amended; and, with gentle exercise, his strength rapidly returned. The parents continued the use of it only three weeks, and at the conclusion of that period the child was perfectly recovered. It is now a year and a half since his indisposition, and from that time to this he has experienced no return of his former complaints, but is a stout, healthy boy.

It was in consequence of witnessing this singular recovery, that a woman, whose child was nearly in the same situation, determined to try the efficacy of the yeast, and in a month the patient was restored, to the inexpressible joy of the mother and surprise of those who had been acquainted with the circumstances. During the administration of the yeast to both, no other medicine was resorted to, nor were they attentive to diet. I never knew it given in such a case before; but as it has been productive of the most happy effects in the two first, I shall not only recommend it to every one, but the first opportunity that occurs shall give it another trial.

The yeast preferred was the newest, because it was not so aperient as the stale, and to both the children it was given by itself; it neither occasioned nausea, flatulence, nor pain.

If you think this information worthy a place in your excellent publication, I shall feel myself honoured by its insertion; and hope some gentleman of extensive practice will observe its influence, and favour the public with his observations.

I have the honour to be,

GENTLEMEN,

Your humble servant,

*Winslow, Nov.* 17, 1799. J. H. GROSE.

